# Reviews and Book Notices

**Published:** 1879-08

**Authors:** 


### Reviews and Book Notices.

ARTICLE XVIII. — A MANUEL OF EXAMINATION OF THE EYES. By Dr. E. Landolt, Directeur-adjoint of the Ophthalmological Laboratory at the Corbonne, Paris. Translated by Swan M. Burnett, M.D., Lecturer on Ophthalmology and Otology in the Medical Department of the University of Georgetown, etc. Revised and Enlarged by the Author. Philadelphia: D. G. Brinton, 115 South Seventh St., 1879; pp. 307.

This book is a most admirable and complete *exposé* of our means and methods of making a thorough scientific examination of the human eye. The scope of the work is delineated on page 20, in the following words: "We shall divide our examinations into the *objective* and *subjective*, the first having for its object to make us acquainted with the eye in a state of functional inactivity, the second to determine the condition of the function of vision.

"The *objective* examination should begin with a general inspection of the patient at a distance, in which we note all that we are able to observe as to his general appearance, and, in particular, the appearance of his eyes, as, for example, their position, etc.

"Afterwards, we examine all parts of the visual apparatus which are accessible to the naked eye, the lids, lachrymal passages, conjunctiva, the form and size of the globe, the cornea, sclerotic, iris, crystalline lens and anterior portion of the vitreous humor. The examination is completed by the determination of the ocular tension.

"In the *subjective* examination, the various functions of the eyes are passed in review, such as movements, refraction and accommodation, acuteness of vision, perception of color, limits of the field of vision, the periphery of the retina.

"Finally, the examination with the ophthalmoscope and the oblique light will show us the condition of the interior of the eye and the dioptric media, and also give us definite ideas regarding the refraction."

It will be observed that the author reserves the ophthalmoscopic inspection, though it forms a part of the *objective* examination, for the end of the examinations. His reasons for so doing are that the ophthalmoscope "always dazzles the eye to a greater or less degree, and renders the functional examination afterward somewhat less satisfactory. Moreover, the ophthalmoscopic examination requires a dark room, and it is better to make at first, all those examinations that are possible by daylight. Moreover, the facts gained by subjective examination are very useful in interpreting the ophthalmoscopic appearances."

This is, perhaps, a matter of opinion rather than of fact; for in our experience we cannot recall a case in which a preceding use of the ophthalmoscope has interfered with the subsequent examination of the functions. But on the other hand, we find it very advantageous to ascertain by the ophthalmoscope the condition of the interior of the eye in all cases of disturbed vision, before we enter into a minute examination of the functions. If the ophthalmoscope reveals a diseased retina as the cause of the visual defect, the functional examination at once is shortened, because it would neither be necessary nor judicious to submit such an eye to a prolonged test of its refraction or accommodation.

In the chapter on "Refraction and Accommodation," the metric system is adopted, according to the proposition of the International Ophthalmological Surgeons held in Brussels in 1875. The unit of the new system is a lens of *one meter focal distance*; it is called a *Dioptry*; and we are informed that the most essential advantages of the new system over the old one are:

1. Numbering the lenses according to their focal power.

2. The unit sufficiently feeble, so that the numbers of the glasses generally in use should be expressed in *whole numbers* and not in fractions.

3. Having the intervals increase gradually from the feebler to the stronger numbers.

This is not the place for discussing the pros and cons of this new departure in optics; but we must say that the reader of Landolt's book may have some difficulty in appreciating the wisdom of this new system with a unit sufficiently feeble to enable us to express the numbers of the lenses in *whole numbers*, if, half a page further on, he is informed that: "It has been found in practice, however, that we have need of lenses weaker than one dioptry. And for this reason there have been admitted into the scale fractions of a dioptry. We have lenses of $\frac{3}{4}$ D. (0.75 D.), $\frac{1}{2}$ D. (0.50 D.) and $\frac{1}{4}$ D. (0.25 D.). Quarters of a dioptry have likewise been introduced between the weaker numbers of the series up to No. 2.5, and half dioptries from No. 2.5 up to No. 6."

But this is no fault of Landolt's, and cannot detract an iota from the merits of his book. Written in the attractive, easy style of lectures, unincumbered by unnecessary mathematical formulas, printed on heavy paper and in large and clear type, translated with care and skill into fluent English, this book will contribute largely toward awakening greater interest for ophthalmology among the reading members of our profession.

F. C. H.

ARTICLE XIX.—A GUIDE TO THERAPEUTICS AND MATERIA MEDICA. By Robert Farquharson, M.D., Edin. Second American edition, revised by the author. Enlarged and adapted to the U. S. Pharmacopœia by Frank Woodbury, M.D. Cloth, pp. 498. 1879. Philadelphia: H. C. Lea.

The special feature about this book is the arrangement in parallel columns of the physiological and therapeutical action of the various drugs considered. The author has felt it necessary to make a quasi-apology for the use of the words "Materia Medica," and we think with reason. We fail to find anything in the book that justifies the interpolation. The value of a new book on Therapeutics must be looked for in the accuracy, conciseness and completeness of the information afforded.

Speaking of ammonia, our authors tell us (p. 130): "It has, therefore, been used with success by Richardson in those cases where, as after delivery, *diphtheria*, *ovariotomy*, etc., a clot is forming in the heart, and he recommends it by injection into the veins, stopping short of the solution of the red corpuscles." We might have been favored with information how to know when a clot is "forming in the heart," how we are to know when to stop "short of the solution of the red corpuscles," and of what strength to make the injection.

Speaking of aconitia, p. 105, we find, "not given internally." On page 109 we read: "Prof. Gubler, of Paris, values it very highly in neuralgia of the fifth nerve, which he has never known to resist a quarter of a milligram of the nitrate of aconitia."

Although the foundation of this book was laid across the water, the English is by no means without blemish. R. T.

ARTICLE XX.—PHOTOGRAPHIC ILLUSTRATIONS OF SKIN DISEASES. By George Henry Fox, A.M., M.D., Clinical Professor of Dermatology, Starling Medical College, Columbus, O., Surgeon to the New York Dispensary, etc.. Forty-eight colored plates taken from life. *Part No.* 1: Comedo, Acne (Vulgaris), Lepra (Tuberosa), Elephantiasis. *Part No.* 2: Keloid, Rosacea, Psoriasis (Nummulata), Ichthyosis (Simplex). *Part No.* 3: Fibroma (Pendulum), Varicella, Zoster (Pectoralis), Zoster (Lumbalis), Eczema (Universale). *Part No.* 4: Leucodermia, Chromophytosis, Favus (Capitis), Favus (Corporis), Eczema (Cruris). New York: E. B. Treat, No. 805 Broadway. Sold by subscription. W. T. Keener, Chicago agent.

Sharply and clearly taken photographs are probably the most perfect representations of nature which it is possible to obtain. They fail, however, in respect to coloring, which is so essential to a faithful portraiture of any object possessing color. Artistically colored photographs give an idea of the objects represented which it is difficult to excel in the matter of accuracy.

These illustrations of skin diseases are admirably perfect representations of the diseases named above, since they meet very faithfully the requirements as to color and exact reproduction of the original. Dr. Fox has been for several years identified with the effort to represent the dermatological field with accurate photographs, and indeed had excellent results even before he attempted to reproduce the pathological tints. In the present work he has received, in the labor of coloring his impressions, the excellent aid of Dr. Gaertner, who was lately a student under the famous Hebra, in the General Hospital of Vienna.

By a method which has never before been applied to photographs of diseases of the skin, the publishers have succeeded in printing directly from the glass photographic negatives, and can thus assure their subscribers that the impressions are indelible.

The letter press which accompanies and explains each photograph, is a concise and clear exposition of the diagnosis and treatment of each disease considered, a feature which will especially commend these illustrations to the general practitioner.

It should be added that the forty-eight illustrations which it is intended to produce will be selected from the author's extensive and valuable collection. There will, therefore, be no delay in the production of the work.

We recommend these photographs to the student and practitioner with the assurance that they are admirably exact in representation, and well calculated to assist in acquiring a knowledge of dermatology. We cannot conclude better than by quoting the words with which Dr. Fox introduces his work: "The study of skin diseases without cases or colored plates, is like the study of osteology without bones, or the study of geography without maps." J. N. H.

ARTICLE XXI. — READING BOOK OF ENGLISH CLASSICS FOR YOUNG PUPILS. Selections from the standard literature of England and America. By C. W. Leffingwell, D.D. New York: S. G. Putnams' Sons, 1879; 12mo., pages, 403.

Any step in the direction of improving the hygiene of the school-room, is worthy of notice in a medical journal. In this reader the compiler has applied correct principles, as regards size of type and tint of paper. If every school book in each department of study were printed with due respect to these principles — if in the preface, or somewhere in the text of these books, were a few rules on the use and abuse of the eyes — much injury of sight, in spite of ignorance and carelessness, would be avoided.

Commissioners who have in charge the construction of school buildings, can do much to preserve good vision in the young, by securing sufficient evenly distributed light, and also by constructing seats and desks in the most approved form.

We trust in future editions of this reader and in other similar works, which the author has in preparation, more will be said on these important subjects. E. L. H.

ARTICLE XXII.—A GUIDE TO THE QUALITATIVE AND QUANTITATIVE ANALYSIS OF THE URINE, DESIGNED FOR PHYSICIANS, CHEMISTS AND PHARMACISTS. By Dr. C. Neubauer, Professor, etc., in Wiesbaden, and Dr. J. Vogel, Professor of Medicine in the Uuiversity at Halle. New York: William Wood & Co, 1879.

This is a translation of the seventh German edition of Neubauer and Vogel's well-known manual of Urinalysis. It is a work which needs no commendation at our hands; it is a noble record of painstaking and untiring industry and scholarship. We are glad to be able to add that the translator (Dr. Elbridge G. Cutler), has done his work wisely and well — so well in fact, that the book, in its English dress, is readable and interesting.

